# Utility of the TG/HDL-C Ratio in Estimating Pediatric Cardiometabolic Risk in a Community Hospital

**DOI:** 10.3390/children11111277

**Published:** 2024-10-23

**Authors:** Adeola O. Awujoola, Ana P. Torga, Jose E. Valencia, Hermella Alemneh, Olaseni P. Prince, Sandipagu P. Kant

**Affiliations:** 1 Department of Pediatric Cardiology, Duke University Hospital, Durham, NC 27710, USA; 2 Children’s National Hospital, Washington, DC 20010, USA; atorga@childrensnational.org; 3 Department of Pediatrics, Bronxcare Health System, 1650 Grand Concourse, New York, NY 10457, USAoprince@bronxcare.org (O.P.P.); skant@bronxleb.org (S.P.K.); 4 Department of Internal Medicine, Washington State University, Everett, WA 98201, USA; hermella.alemneh@wsu.edu

**Keywords:** metabolic syndrome, cardiovascular disease, triglyceride, high-density lipoprotein cholesterol

## Abstract

**Background**: The evaluation of metabolic syndrome (MetS) is critical among children and adolescents as it can predict cardiovascular disease in adulthood. The TG/HDL-C ratio has the best predictive accuracy among the different lipid ratios. This study aims to evaluate the prevalence and factors associated with a high TG/HDL-C ratio and the predictive accuracy for pediatric MetS. **Methods**: This is a cross-sectional study of adolescents aged 9–21 to determine the prevalence of and associations between an elevated TG/HDL-C ratio and MetS. A ROC curve analysis was performed to determine the predictive accuracy of TG/HDL-C with MetS. **Results**: Of the 604 subjects, 29.8% had elevated TG/HDL-C, MetS was identified in 28.2%, and 54.5% were obese. Among patients with MetS, those with obesity were more likely to have an elevated TG/HDL-C ratio compared to those with a normal BMI (64% vs. 14%, *p* < 0.05). Gender, obesity, ethnicity, and METS are significantly associated with a high TG/HDL-C ratio. TG/HDL-C has a good discriminatory ability to distinguish patients with MetS from those without (AUC 0.65, *p* < 0.05). **Conclusions**: The TG/HDL-C ratio was able to distinguish between children and adolescents with MetS. Hence, the TG/HDL-C ratio may be used in the rapid risk assessment of pediatric MetS, especially in those with obesity.

## 1. Introduction

Childhood metabolic syndrome (MetS) is defined as a constellation of risk factors for cardiovascular diseases (CVDs) and type 2 diabetes mellitus [1,2,3,4]. There has been a consistently increasing prevalence of overweight and obesity among children and adolescents in the last decade. Concomitantly, an increased incidence in early onset MetS or the clustering of cardiometabolic risk factors has been seen [5]. Because childhood MetS often leads to adult MetS, early detection and management of pediatric MetS is critical for preventing CVD in adulthood [6]. CVD is the leading cause of death in the adult population. However, the central event (atherosclerosis) that propagates this event has its onset in early childhood [7,8,9].

The lack of a universally accepted definition of pediatric MetS has hampered diagnostic efforts, and several studies have been performed to compare criteria without any consensus [10,11,12]. The definitions of MetS in children and adolescents are more varied than those used in adults, and there are currently more than 40 different definitions proposed [13]. The different definitions for MetS do not classify the same individual as having MetS, and the prevalence varies between diagnostic approaches. Resultantly, the American Heart Association has called for further research focusing on tools that can identify children who are most at risk for cardiometabolic disease rather than finding a universal definition for MetS [2,14].

The evaluation of cardiometabolic risk is critical, especially among obese children and adolescents. Given the difficulty in routinely aggregating all the components for diagnosing MetS, recent literature has looked at the lipid ratios’ utility in detecting MetS. Studies have shown that the triglyceride/high-density lipoprotein cholesterol ratio (TG/HDL-C) is a more reliable surrogate for predicting MetS and cardiometabolic risk compared to other lipid ratios like the total cholesterol/HDL-C (TC/HDL-C) ratio, the low-density lipoprotein cholesterol (LDL-C/HDL-C) ratio, and non-HDL-C/HDL-C, with a high predictive accuracy [15,16,17,18,19,20,21,22]. Although there is no consensus about the cut-off value for the TG/HDL-C ratio, existing literature has shown increased cardiometabolic risk for ratios between two and three among children and adolescents, with sensitivity and specificity comparable to the criteria currently proposed to diagnose metabolic syndrome [17,20,23].

We conducted a pilot cross-sectional analysis of children aged 9–21 years from the National Health and Nutrition Examination Survey (NHANES) 2011–2018 to evaluate the prevalence of high TG/HDL-C among US adolescents [24]. Our study showed an elevated TG/HDL ratio of 27.5%. This makes it more pertinent to evaluate cardiometabolic risk among our population in the Bronx (New York), with many at-risk children that will benefit from the early identification of risk and hence prompt the initiation of intervention. The utility of the TG/HDL ratio for cardiometabolic risk estimation has been well established in the adult population [16,25,26,27,28]. Meanwhile, its value in the pediatric population has been sparsely investigated [27,29,30]. This will be one of the few studies in the US to explore the utility of a lipid ratio for MetS identification among children and adolescents. Our study explored the risk identification among these groups to estimate their risk of cardiometabolic disorder and provide a tool for rapid risk assessment. Furthermore, having an objective estimate of the burden of cardiometabolic risk among our vulnerable, underserved population will provide more evidence for the targeted intervention that can preclude a future of unhealthy young adults. Hence, our study aims to evaluate the prevalence of high TG/HDL-C, examine factors associated with an elevated TG/HDL-C ratio, and determine the predictive index of utilizing TG/HDL-C in predicting metabolic syndrome among children and adolescents at our center.

## 2. Materials and Methods

Study Design and Sample Size: We performed a retrospective chart review for children and adolescents who were seen in our pediatric outpatient unit over one year. For adequate study power, we aimed to enroll as many subjects as possible that would qualify for inclusion. The estimated sample size to determine a disease prevalence of 25% with a 99% confidence level is *n* = 455, which approximates 500, to account for incomplete abstraction that might make some entries invalid. To account for unforeseen incomplete abstractions or missing data, we utilized a final sample size of 604.

*Study Population:* South Bronx is a medically underserved area in New York, serving a diverse population that includes Hispanics, non-Hispanic Blacks (African Americans, immigrants from Africa), and other immigrants from Southeast Asia. The pediatric population in this community has one of the highest prevalences of obesity in the United States [31]. Our study focused on children and adolescents between 9–21 years of age, seen as a BronxCare Health System pediatric outpatient between January to December 2021. We excluded children with congenital heart conditions, type 1 diabetes mellitus, hypothyroidism, metabolic disorders affecting lipid metabolism, and those taking medications influencing weight. This study was approved by the BronxCare Health System institutional review board (IRB) by expedited review with IRB # 01132202.

*Measurement Variables:* Biochemical parameters, which included the lipid profile, triglycerides (TG), high-density lipoprotein cholesterol (HDL-C), low-density lipoprotein cholesterol (LDL-C), total cholesterol (TC), hemoglobin A1C (HbA1C), and liver enzymes (aspartate aminotransferase (AST), alanine aminotransferase (ALT), from patient encounters were extracted from electronic medical records (EMR) and included in the study. Lipid profiles are routinely checked in all adolescent patients at 9–11 years and 17–21 years, per American Academy of Pediatrics (AAP) guidelines, while other patients with comorbidities or other risk factors receive additional blood tests.

The primary outcome variable is the TG/HDL-C ratio. Studies have employed different cut-offs. Most studies [18,19], with demographics similar to ours, identified 2.0 or 3.0 as the cut-off value at which sensitivity was optimized without compromising the specificity. We used a cut-off of ≥2.5 in our study to determine “high” versus “low” levels. For MetS diagnosis, the secondary outcome variable, clusters of cardiometabolic risk factors were utilized. A study by Salazar et al. showed that the relationship between body mass index (BMI) and waist circumference with MetS and its components are comparable. A study by Quijada et al. [22] demonstrated that the TG/HDL-C ratio and BMI significantly explained MetS. In this study, MetS was defined by clustering three or more cardiometabolic risk factors, which include BMI > 95th percentile, triglyceride > 110 mg/dL, HDL-C < 40 mg/dL, systolic blood pressure > 90th percentile, and fasting blood sugar ≥ 100 mg/dL.

*Covariates:* Demographic information (i.e., age, sex, ethnicity) was also extracted from EMR. Age was recorded as a categorical variable with three subcategories: 9–11 years (prepubertal), 12–16 years (pubertal), and 17–21 years (post-pubertal). Sex was recorded as male or female. The self-reported or documented ethnic identifications were recategorized into non-Hispanic blacks, Hispanics, and others. Clinical parameters like systolic and diastolic blood pressure and BMI (kg/m^2^) were recorded as continuous variables. BMI was further categorized into normal weight (5th–84th percentile), overweight (85th–94th percentile), and obese (>95th percentile). Blood pressures were categorized as greater or less than the 90th percentile.

*Statistical Analysis:* A descriptive analysis was performed to describe the baseline characteristics. The categorical variables were presented as frequencies and percentages, while the continuous variables were presented as the mean or median. A bivariate analysis was used to examine if an elevated TG/HDL-C ratio and MetS status significantly differed among the study participants based on baseline and biochemical characteristics. Categorical variables were analyzed using chi-square statistics. The continuous variables were analyzed with Student’s *t*-test if the data were normally distributed or with the Mann–Whitney U or Kruskal–Wallis test if the data were not normally distributed. Additionally, we stratified the data based on obesity status and we examined the association between the TG/HDL-C ratio and MetS using the chi-square test. The variables with significant findings in the bivariate analyses were modeled with multivariate logistic regression analysis to determine the factors associated with an elevated TG/HDL-C ratio. Additionally, to determine the clinical utility of the TG/HDL-C ratio for identifying patients with MetS, a receiver operating characteristic (ROC) curve analysis was performed to evaluate how well the TG/HDL-C ratio could discriminate between individuals with and without MetS. Statistical significance for all analyses was set at *p*-value < 0.05. All statistical analyses were performed with SAS 9.40 software.

## 3. Results

### 3.1. Baseline Characteristics of Subjects

Of the total 604 subjects included in the final analysis, 51% were male, 54.5% were in the age group of 12–16 years (with a median age of 14 years), and 46% reported or were documented as Hispanic. The prevalence of obesity and overweight in the study population was 55% and 18%, respectively. The prevalence of a high TG/HDL-C ratio and MetS were 29.8% and 28%, respectively. The individual components of MetS (elevated triglyceride, low HDL-C, and systolic blood pressure > 90th percentile), were all in the range of 20–30%, with obesity (54.5%) constituting the most prevalent abnormality (Table 1).

### 3.2. Characteristics of Subjects Grouped by TG/HDL-C or MetS

Table 2 compares the characteristics of subjects based on the TG/HDL-C ratio and MetS status using bivariate analysis. Except for blood pressure, other characteristics were similarly distributed among the TG/HDL-C group and the MetS group. More males had an elevated TG/HDL-C ratio compared to females (62.2% versus 37.8%, *p* < 0.05). Similarly, more males were classified as having MetS compared to females, but the difference was not statistically significant. Age group was not associated with either elevated TG/HDL or MetS. In terms of ethnic background, elevated TG/HDL-C ratios and MetS were seen more in Hispanics compared to the non-Hispanic blacks (56% versus 26.1%, *p* < 0.05) and (50% versus 35.3%, *p* = 0.07), respectively. Compared to normal weight and overweight subjects, those with obesity had a higher prevalence of elevated TG/HDL-C (73.7%, *p* < 0.01) and MetS (75.3%, *p* < 0.01). For the lipid profile components, subjects with elevated triglyceride (78.9% vs. 21.1%; *p* < 0.01) and low HDL-C (58.3% vs. 41.7%; *p* < 0.01) had an elevated TG/HDL-C ratio compared to those with a normal lipid profile. Similarly, in the MetS versus no MetS group, most subjects with MetS had elevated triglyceride levels and low HDL-C levels. Meanwhile, fewer patients with a blood pressure percentile > 90th (27.2 vs. 72.8%; *p* < 0.03) had an elevated TG/HDL-C ratio. Contrastingly, the majority (76.5%) of subjects with a blood pressure (BP) > 90th percentile had MetS. The prevalence of elevated non-HDL-C (>145 mg/dL) was 11.4% and positively correlated with the TG/HDL-C ratio (r = 0.49, *p* < 0.05).

Following stratification based on obesity status, among subjects with obesity that had MetS, 64% had an elevated TG/HDL ratio compared to 14% among MetS patients without obesity (*p* < 0.05) (Table 3). Using a cut-off value of 2.5, the sensitivity of the TG/HDL-C ratio for identifying MetS was 52% and the specificity was 79%. However, following stratification based on obesity status, the sensitivity improved to 64% and the specificity improved to 76%.

### 3.3. Factors Associated with Elevated TG/HDL-C

Table 4 shows factors associated with an elevated TG/HDL-C ratio following multivariate logistic regression analysis. The odds of an elevated TG/HDL-C ratio are 3.4 times higher among patients with MetS compared to those without MetS (aOR 3.41 95%, CI 2.25–5.18). Obese children were three times more likely to have an elevated TG/HDL-C ratio compared to their normal weight counterparts (aOR 3.02 95%, CI 1.77–5.12). The odds of an elevated TG/HDL-C ratio among females were reduced by approximately 54% compared to males (aOR 0.46 95%, CI 0.31–0.69). The TG/HDL-C, at a cut-off of 2.5, had a good discriminatory ability to distinguish patients with MetS from those without MetS, with an area under the curve (AUC) value of 0.6528 (*p* < 0.05). 

## 4. Discussion

Cardiovascular disease (CVD) is the leading cause of mortality and morbidity among adults. Atherosclerosis, a central event in this process, starts in childhood [7,8,9]. Hence, the need for tools that can easily screen for cardiometabolic risk in the childhood/adolescent period is required. Metabolic syndrome has been identified as a good predictor of CVD risk. However, it is cumbersome to put its components together, and there is no consensus on its diagnostic criteria to date. The clinical utility of an elevated TG/HDL ratio as a predictive tool has been the subject of recent scientific ventures. In our study, the prevalence of an elevated TG/HDL-C and MetS were 29.8% and 28%, respectively. Our study showed that among patients with MetS, a significantly higher number of obese patients had elevated TG/HDL ratios compared to non-obese patients. An elevated TG/HDL-C ratio had a good discriminatory ability to distinguish patients with MetS from those without MetS (AUC 0.6528, *p* < 0.05).

According to many experts, the increasing burden of obesity is the driving force for the high prevalence of MetS and other cardiometabolic risks [1,2]. In our local analysis, the prevalence of an elevated TG/HDL-C ratio is high at about 30%, which was similar to the 28% obtained in our prior NHANES (2011–2018) dataset analysis [24]. This finding is consistent with that of previous studies [3,21,25]. Similarly, the high prevalence of MetS (28%) in our study is consistent with previous studies among diverse racial groups [23,31,32]. The variation in the prevalence of MetS is not unexpected given the varying definitions of MetS adopted among the studies. In our general study population, the sensitivity and specificity of TG/HDL-C for MetS diagnosis were 52% and 79%, respectively. Following stratification by obese status, sensitivity among the obese group improved to 64% with no huge sacrifice in specificity at 76%. Meanwhile, among the non-obese group, the sensitivity and specificity were 14.3% and 82%, respectively. These findings suggest that the TG/HDL-C ratio may be of greater utility as a screening tool among obese adolescents, which is consistent with findings in other studies [3,19,22,27,32,33]. This supports the previously reported association between obesity and increased cardiometabolic risk [2,27,34].

The TG/HDL-C ratio sensitivity seems to vary among different racial/ethnic groups. This is consistent with the findings of the multiethnic study by Giannini et al., which included Hispanic, African American, and Caucasian subjects, where they proposed the need for different cut-offs for the TG/HDL-C ratio [19]. It is of note that in our population, Hispanics had a higher prevalence of an elevated TG/HDL-C ratio and MetS. Studies among similar populations showed similar results. For instance, a study from a Hispanic school population outside the US reported that a higher percentage of their study subjects with obesity, hypertension, and MetS had an elevated TG/HDL-C ratio [22]. Another study found a high correlation between the TG/HDL-C ratio and insulin resistance in indigenous Argentinian children [35]. On the other hand, Sumner et al. found the TG/HDL-C ratio to have a very low sensitivity for identifying insulin resistance in African American adults [36]. Further analysis using race/ethnicity-specific cut-off values may be valuable for accurate risk identification.

Comparison of the baseline characteristics based on the TG/HDL-C ratio and MetS status showed similar distributions among the different variables in both groups. This finding reinforced the utility of TG/HDL-C for presumptive MetS diagnosis and is similar to findings by Shou-Yu et al. [30], where a strong relationship between the MetS components and TG/HDL-C was found. Our study found that an elevated TG/HDL-C ratio is associated with MetS (*p* < 0.05) and can be a predictive tool in identifying MetS (AUC 0.6528). This finding further supports that the TG/HDL-C ratio may be a simple and rapid risk assessment tool for pediatric MetS, hence leading to prompt identification of at-risk children and adolescents. This is consistent with other studies performed to evaluate the association of this lipid ratio with the other associated risk factors of MetS [16,21,27,30].

There are limitations to our study. Firstly, because we used a cross-sectional design, we can only suggest an association between TG/HDL-C and pediatric MetS and cannot distinctly establish risk/causation. Hence, further studies, preferably prospective, will be required. Secondly, the predominance of non-fasting lipid parameters might have underestimated the findings. To address this, we assessed our cohort’s non-HDL-C levels, which are positively correlated with the TG/HDL-C ratio. Also, variables such as diet, physical activity, comorbidities, and pharmacotherapy data were not included in the analysis because they were not consistently gathered during the encounters, and not retrievable during the chart review.

Furthermore, since our findings with the TG/HDL-C ratio are consistent with previous studies that established the ratio’s significant association with MetS, this means that misclassification bias, if present, did not blur the association; if anything, we would anticipate a stronger association. Lastly, since the study population is primarily of Hispanic and non-Hispanic black origin, our findings are not necessarily relevant to other racial groups.

## 5. Conclusions

Our patients have a disproportionately high prevalence of elevated TG/HDL ratios and obesity compared to the national average. Hispanic males with obesity may be at the greatest risk of CVDs. TG/HDL-C has a good discriminatory ability to distinguish patients with and without MetS. Our findings support previous evidence of a good correlation between TG/HDL and MetS. Hence, TG/HDL may be employed for rapid risk assessment. A high-quality prospective study should be conducted to validate these findings.

## Figures and Tables

**Table 1:** Summary of baseline characteristics.

Variable	Frequency	Percentage
**Age group**		
9–11	173	28.6
12–16	329	54.5
17–21	102	16.9
Age, median (IQR)	14 (11–17)	
**Gender**		
Male	310	51.3
Female	294	48.7
**Ethnicity**		
Non-Hispanic Black	255	42.2
Hispanics	278	46
Others	71	11.8
**BMI**		
Normal weight	162	27.3
Overweight	108	18.2
Obese	324	54.5
Median (IQR)	27.2 (21.8–32.5)	
**Triglyceride**		
>110 mg/dL	165	27.3
<110 mg/dL	439	72.7
Median (IQR), mg/dL	80 (58–113)	
**HDL-C**		
>40 mg/dL	445	73.7
<40 mg/dL	159	26.3
Median (IQR), mg/dL	46 (40–56)	
**Other Lipid Components**		
Cholesterol Median (IQR), mg/dL	154 (136–174)	
LDL Median (IQR), mg/dL	85 (71–105)	
Non-HDL-C Median (IQR), mg/dL	104 (87–125)	
**TG/HDL-C**		
>2.5	180	29.8
<2.5	424	70.2
Median (IQR)	1.67 (1.14–2.85)	
**Systolic blood pressure**		
>90th percentile	130	21.5
<90th percentile	474	78.5
Median (IQR), mg/dL	114 (108–120)	
**Metabolic syndrome**		
Yes	170	28.2
No	434	71.9

Descriptive analysis—categorical variables are presented as *n* (%). Continuous variables are presented as the median (25%, 75%) for skewed data. HDL-C—high-density lipoprotein cholesterol, TG—triglycerides, TG/HDL-C—ratio of TG and HDL-C, non-HDL-C—non-high-density lipoprotein cholesterol, BMI—Body mass index.

**Table 2:** Comparing demographic and cardiometabolic risk factors based on the TG/HDL-C ratio and METs status.

Variable	TG/HDL-C Ratio		*p*-Value	METs		*p*-Value
	≥2.5	<2.5		Yes	No	
**Age group**			0.38			0.38
9–11	45 (25)	128 (30.2)		53 (31.20)	120 (27.7)	
12–16	101 (56.1)	228 (53.8)		85 (50)	244 (56.2)	
17–21	34 (18.9)	68 (16)		32 (18.8)	70 (16.3)	
**Gender**			<0.001 *			0.89
Male	112 (62.2)	198 (46.7)		88 (51.8)	222 (51.2)	
Female	68 (37.8)	226 (53.3)		82 (48.2)	212 (48.9)	
**Ethnicity**			<0.001 *			0.07 *
NHB	47 (26.1)	208 (49.1)		60 (35.3)	195 (44.9)	
Hispanics	102 (56.7)	176 (41.5)		85 (50)	193 (44.5)	
Others	31 (17.2)	40 (9.4)		25 (14.7)	46 (10.6)	
**BMI**			<0.001 *			<0.001 *
Normal weight	22 (12.6)	140 (33.4)		20 (11.7)	142 (33.5)	
Overweight	24 (13.7)	84 (20.1)		22 (12.9)	86 (20.3)	
Obese	129 (73.7)	195 (46.5)		128 (75.3)	196 (46.2)	
**Triglyceride**			<0.001 *			<0.001 *
>110 mg/dL	142 (78.9)	23 (5.4)		86 (50.6)	79(18.2)	
<110 mg/dL	38 (21.1)	401 (94.6)		84 (49.4)	355 (81.8)	
**HDL-C**			<0.001 *			<0.001 *
>40 mg/dL	75 (41.7)	370 (87.3)		92 (54.1)	353 (81.3)	
<40 mg/dL	105 (58.3)	54 (12.7)		78 (45.9)	81 (18.7)	
**Systolic blood pressure**			0.03 *			<0.001 *
>90th percentile	49 (27.2)	81 (19.1)		130 (76.5)	0	
<90th percentile	131 (72.8)	343 (80.9)		40 (23.5)	434 (100)	
**Metabolic syndrome**			<0.001 *			
Yes	88 (48.9)	82 (19.3)				
No	92 (51.1)	342 (80.7)				

Bivariate analysis—Comparing demographic and cardiometabolic risk factors based on the TG/HDL-C ratio and METs status. Results of categorical data are presented as percentages and frequency. Pearson’s Chi-Square was used to compare differences between the baseline characteristics among the children and adolescents based on their TG/HDL-C and METs status. * Indicates statistical significance (*p* < 0.1). HDL-C—high-density lipoprotein cholesterol, TG—triglycerides, TG/HDL-C—ratio of TG and HDL-C, BMI—body mass index, METs—metabolic syndrome.

**Table 3:** (**a**) Association between the TG/HDL-C ratio and METs. (**b**) Stratified by obesity status: association between the TG/HDL-C ratio and METs.

**(a)**					
**TG/HDL-C Ratio**	**METs**				***p*-Value**
	**Yes**		**No**		
≥2.5	88 (51.8)		92 (21.2)		<0.001 *
<2.5	82 (48.2)		342 (78.8)		
**(b)**					
**TG/HDL-C Ratio**	**Obese**		**Not Obese**		***p*-Value**
	**METs**	**No METs**	**METs**	**No METs**	
≥2.5	82 (64.1)	47 (24.0)	6 (14.3)	40 (17.5)	<0.001 *
<2.5	46 (35.9)	149 (76.0)	36 (85.7)	188 (82.5)	

Bivariate analysis: (a) unstratified, (b) stratified by obese status—Association between the TG/HDL-C ratio and METs. Results of categorical data are presented as percentages and frequency. Pearson’s Chi-Square was used to compare differences between the dichotomized TG/HDL-C ratios among children and adolescents based on their METs status. * Indicates statistical significance (*p* < 0.1).

**Table 4:** Factors associated with an elevated TG/HDL ratio.

Variable	Adjusted Odd Ratio	95% Confidence Interval	
		Lower Interval	Upper Interval
**Female**	0.462	0.308	0.691 *
**Age group**			
Pubertal	Reference		
Prepubertal	0.713	0.447	1.137
Postpubertal	1.214	0.712	2.073
**Ethnicity**			
Others	Reference		
NHB	0.366	0.236	0.568 *
Hispanics	1.098	0.612	1.971
**BMI**			
Normal	Reference		
Overweight	1.765	0.903	3.450
Obese	3.024	1.765	5.181 *
METs present	3.412	2.249	5.176 *

Factors associated with an elevated TG/HDL ratio. Adjusted OR are presented. Adjusted OR < 1 denotes factors that are associated with a lesser likelihood of an elevated TG/HDL ratio while OR > 1 denotes factors that are associated with a higher likelihood of an elevated TG/HDL ratio. Hosmer–Lemeshow test was performed (*p* = 0.1751), which indicated that our models fit the data. Max-rescaled R-square (R^2^ = 0.2542) was used to assess how well our model explained the variability in the TG/HDL ratio. * Indicates statistical significance, which is indicated by a 95% confidence interval that excludes the null value of 1.

## Data Availability

The datasets generated during and/or analyzed during the current study are available upon reasonable request from the corresponding author due to privacy and legal reasons.

## References

[1] Cleeman J.I. (2001). Executive summary of the third report of the National Cholesterol Education Program (NCEP) expert panel on detection, evaluation, and treatment of high blood cholesterol in adults (adult treatment panel III). J. Am. Med. Assoc..

[2] Grundy S.M., Cleeman J.I., Daniels S.R., Donato K.A., Eckel R.H., Franklin B.A., Gordon D.J., Krauss R.M., Savage P.J., Smith S.C. (2005). Diagnosis and management of the metabolic syndrome: An American Heart Association/National Heart, Lung, and Blood Institute scientific statement. Circulation.

[3] Lee A.M., Gurka M.J., DeBoer M.D. (2016). Trends in metabolic syndrome severity and lifestyle factors among adolescents. Pediatrics.

[4] Rodrigues A.N., Abreu G.R., Resende R.S., Goncalves W.L., Gouvea S.A. (2013). Cardiovascular risk factor investigation: A pediatric issue. Int. J. Gen. Med..

[5] Tropeano A., Corica D., Pomi A.L., Pepe G., Morabito L.A., Curatola S.L., Casto C., Aversa T., Wasniewska M. (2021). The metabolic syndrome in pediatrics: Do we have a reliable definition? A systematic review. Eur. J. Endocrinol..

[6] Morrison J.A., Friedman L.A., Wang P., Glueck C.J. (2008). Metabolic Syndrome in Childhood Predicts Adult Metabolic Syndrome and Type 2 Diabetes Mellitus 25 to 30 Years Later. J. Pediatr..

[7] Berenson G.S., Srinivasan S.R., Bao W., Newman W.P., Tracy R.E., Wattigney W.A. (1998). Association between Multiple Cardiovascular Risk Factors and Atherosclerosis in Children and Young Adults. N. Engl. J. Med..

[8] O’Leary D.H., Polak J.F., Kronmal R.A., Manolio T.A., Burke G.L., Wolfson S.K. (1999). Carotid-Artery Intima and Media Thickness as a Risk Factor for Myocardial Infarction and Stroke in Older Adults. N. Engl. J. Med..

[9] Hodis H.N., Mack W.J., LaBree L., Selzer R.H., Liu C.-R., Liu C.-H., Azen S.P. (1998). The role of carotid arterial intima—Media thickness in predicting clinical coronary events. Ann. Intern. Med..

[10] Golley R.K., Magarey A.M., Steinbeck K.S., Baur L.A., Daniels L.A. (2006). Comparison of metabolic syndrome prevalence using six different definitions in overweight pre-pubertal children enrolled in a weight management study. Int. J. Obes..

[11] Reuter C.P., Burgos M.S., Barbian C.D., Renner J.D.P., Franke S.I.R., de Mello E.D. (2018). Comparison between different criteria for metabolic syndrome in schoolchildren from southern Brazil. Eur. J. Pediatr..

[12] Martino F., Pannarale G., Puddu P.E., Colantoni C., Zanoni C., Martino E., Torromeo C., Paravati V., Perla F.M., Barilla F. (2015). Is it possible a new definition of metabolic syndrome in childhood?. Eur. Rev. Med. Pharmacol. Sci..

[13] Reisinger C., Nkeh-Chungag B.N., Fredriksen P.M., Goswami N. (2021). The prevalence of pediatric metabolic syndrome—A critical look on the discrepancies between definitions and its clinical importance. Int. J. Obes..

[14] Steinberger J., Daniels S.R., Eckel R.H., Hayman L., Lustig R.H., McCrindle B., Mietus-Snyder M.L. (2009). Progress and Challenges in Metabolic Syndrome in Children and Adolescents A Scientific Statement From the American Heart Association Atherosclerosis, Hypertension, and Obesity in the Young Committee of the Council on Cardiovascular Disease in the Young. Circulation.

[15] Rezapour M., Shahesmaeili A., Hossinzadeh A., Zahedi R., Najafipour H., Gozashti M.H. (2018). Comparison of Lipid Ratios to Identify Metabolic Syndrome. Arch. Iran. Med..

[16] Gasevic D., Frohlich J., Mancini G.J., Lear S.A. (2014). Clinical usefulness of lipid ratios to identify men and women with metabolic syndrome: A cross-sectional study. Lipids Health Dis..

[17] Di Bonito P., Moio N., Scilla C., Cavuto L., Sibilio G., Sanguigno E., Forziato C., Saitta F., Iardino M.R., Di Carluccio C. (2012). Usefulness of the high triglyceride-to-HDL cholesterol ratio to identify cardiometabolic risk factors and preclinical signs of organ damage in outpatient children. Diabetes Care.

[18] Di Bonito P., Valerio G., Grugni G., Licenziati M., Maffeis C., Manco M., del Giudice E.M., Pacifico L., Pellegrin M., Tomat M. (2015). Comparison of non-HDL-cholesterol versus triglycerides-to-HDL-cholesterol ratio in relation to cardiometabolic risk factors and preclinical organ damage in overweight/obese children: The CARITALY study. Nutr. Metab. Cardiovasc. Dis. NMCD.

[19] Giannini C., Santoro N., Caprio S., Kim G., Lartaud D., Shaw M., Pierpont B., Weiss R. (2011). The triglyceride-to-HDL cholesterol ratio: Association with insulin resistance in obese youths of different ethnic backgrounds. Diabetes Care.

[20] Murguía-Romero M., Jiménez-Flores J.R., Sigrist-Flores S.C., Espinoza-Camacho M.A., Jiménez-Morales M., Piña E., Méndez-Cruz A.R., Villalobos-Molina R., Reaven G.M. (2013). Plasma triglyceride/HDL-cholesterol ratio, insulin resistance, and cardiometabolic risk in young adults. J. Lipid Res..

[21] Hermans M.P., Ahn S.A., Rousseau M.F. (2010). log(TG)/HDL-C is related to both residual cardiometabolic risk and β-cell function loss in type 2 diabetes males. Cardiovasc. Diabetol..

[22] Quijada Z., Paoli M., Zerpa Y., Camacho N., Cichetti R., Villarroel V., Arata-Bellabarba G., Lanes R. (2008). The triglyceride/HDL-cholesterol ratio as a marker of cardiovascular risk in obese children; association with traditional and emergent risk factors. Pediatr. Diabetes.

[23] Salazar M.R., Carbajal H.A., Espeche W.G., Sisnieguez C.E.L., Balbín E., Dulbecco C.A., Aizpurúa M., Marillet A.G., Reaven G.M. (2012). Relation among the plasma triglyceride/high-density lipoprotein cholesterol concentration ratio, insulin resistance, and associated cardio-metabolic risk factors in men and women. Am. J. Cardiol..

[24] Awujoola A., Torga P.A., Valencia J., Kant S. Prevalence of Cardiometabolic Risk among US children and Adolescents using TG/HDL-C ratio: Are there differences based on age subgroups?. Proceedings of the Joint Conference of Eastern Society for Pediatric Research (ESPR).

[25] Salazar M.R., Carbajal H.A., Espeche W.G., Aizpurúa M., Sisnieguez C.E.L., March C.E., Balbín E., Stavile R.N., Reaven G.M. (2013). Identifying cardiovascular disease risk and outcome: Use of the plasma triglyceride/high-density lipoprotein cholesterol concentration ratio versus metabolic syndrome criteria. J. Intern. Med..

[26] Gharipour M., Sadeghi M., Nezafati P., Dianatkhah M., Sarrafzadegan N. (2019). Cardiovascular Disease Risk Assessment: Triglyceride/High-Density Lipoprotein versus Metabolic Syndrome Criteria. J. Res. Health Sci..

[27] Iwani A.K.N.Z., Jalaludin M.Y., Zin R.M.W.M., Fuziah Z., Hong J.Y.H., Abqariyah Y., Mokhtar A.H., Mohamud W.N.W. (2019). TG : HDL-C Ratio Is a Good Marker to Identify Children Affected by Obesity with Increased Cardiometabolic Risk and Insulin Resistance. Int. J. Endocrinol..

[28] Baez-Duarte B.G., Zamora-Ginez I., González-Duarte R., Torres-Rasgado E., Ruiz-Vivanco G., Pérez-Fuentes R., The Multidisciplinary Research Group Of Diabetes Celis (2017). Triglyceride/high-density lipoprotein cholesterol (TG/HDL-C) index as a reference criterion of risk for metabolic syndrome (MetS) and low insulin sensitivity in apparently healthy subjects. Gac. Medica De Mex..

[29] Radetti G., Grugni G., Lupi F., Fanolla A., Caroli D., Bondesan A., Sartorio A. (2022). High Tg/HDL-Cholesterol Ratio Highlights a Higher Risk of Metabolic Syndrome in Children and Adolescents with Severe Obesity. J. Clin. Med..

[30] Chu S.-Y., Jung J.-H., Park M.-J., Kim S.-H. (2019). Risk assessment of metabolic syndrome in adolescents using the triglyceride/high-density lipoprotein cholesterol ratio and the total cholesterol/high-density lipoprotein cholesterol ratio. Ann. Pediatr. Endocrinol. Metab..

[31] Ambachew S., Endalamaw A., Worede A., Tegegne Y., Melku M., Biadgo B. (2020). The Prevalence of Metabolic Syndrome in Ethiopian Population: A Systematic Review and Meta-analysis. J. Obes..

[32] Kelishadi R., Hovsepian S., Djalalinia S., Jamshidi F., Qorbani M. (2016). A systematic review on the prevalence of metabolic syndrome in Iranian children and adolescents. J. Res. Med. Sci. Off. J. Isfahan Univ. Med. Sci..

[33] Liang J., Fu J., Jiang Y., Dong G., Wang X., Wu W. (2015). TriGlycerides and high-density lipoprotein cholesterol ratio compared with homeostasis model assessment insulin resistance indexes in screening for metabolic syndrome in the chinese obese children: A cross section study. BMC Pediatr..

[34] Kim J., Lee I., Lim S. (2017). Overweight or obesity in children aged 0 to 6 and the risk of adult metabolic syndrome: A systematic review and meta-analysis. J. Clin. Nurs..

[35] Hirschler V., Maccallini G., Sanchez M., Gonzalez C., Molinari C. (2015). Association between triglyceride to HDL-C ratio and insulin resistance in indigenous Argentinean children. Pediatr. Diabetes.

[36] Sumner A.E., Finley K.B., Genovese D.J., Criqui M.H., Boston R.C. (2005). Fasting triglyceride and the triglyceride-HDL cholesterol ratio are not markers of insulin resistance in African Americans. Arch. Intern. Med..

